# Klotho increases antioxidant defenses in astrocytes and ubiquitin–proteasome activity in neurons

**DOI:** 10.1038/s41598-023-41166-6

**Published:** 2023-09-12

**Authors:** Ana Maria Orellana, Caio Henrique Mazucanti, Leticia Pavan dos Anjos, Larissa de Sá Lima, Elisa Mitiko Kawamoto, Cristoforo Scavone

**Affiliations:** 1 Laboratory of Molecular Neuropharmacology, Department of Pharmacology, Institute of Biomedical Sciences ICB-1, University of São Paulo, Avenida Professor Lineu Prestes, 1524, São Paulo, São Paulo 05508-900 Brazil; 2 Laboratory of Clinical Investigation, Diabetes Section, National Institute on Aging (NIH/NIA), Baltimore, MD USA; 3 Laboratory of Molecular and Functional Neurobiology, Institute of Biomedical Science, University of São Paulo, São Paulo, Brazil

**Keywords:** Neuroscience, Cognitive ageing

## Abstract

Klotho is an antiaging protein, and its levels decline with age and chronic stress. The exogenous administration of Klotho can enhance cognitive performance in mice and negatively modulate the Insulin/IGF1/PI3K/AKT pathway in terms of metabolism. In humans, insulin sensitivity is a hallmark of healthy longevity. Therefore, this study aimed to determine if exogenous Klotho, when added to neuronal and astrocytic cell cultures, could reduce the phosphorylation levels of certain insulin signaling effectors and enhance antioxidant strategies in these cells. Primary cell cultures of cortical astrocytes and neurons from mice were exposed to 1 nM Klotho for 24 h, with or without glucose. Klotho decreased pAKT and mTOR levels. However, in astrocytes, Klotho increased FOXO-3a activity and catalase levels, shielding them from intermediate oxidative stress. In neurons, Klotho did not alter FOXO-3 phosphorylation levels but increased proteasome activity, maintaining lower levels of PFKFB3. This study offers new insights into the roles of Klotho in regulating energy metabolism and the redox state in the brain.

## Introduction

The Klotho protein was first identified in 1997, when animal studies linked its absence to an accelerated aging phenotype. Consequently, Klotho has been recognized as an anti-aging protein^1,2^. Within the central nervous system (CNS), the choroid plexus is regarded as the primary site of Klotho synthesis^3,4^. However, it is also found in lower concentrations in other regions, including the cortex, hippocampus, cerebellum, striatum, substantia nigra, olfactory bulb, and medulla^5–8^.

Klotho protein exists in two primary isoforms: membrane and soluble. The membrane Klotho comprises two domains, KL1 and KL2. These domains can be cleaved, resulting in the primary soluble forms found in circulation^3^ and in the cerebrospinal fluid. These forms exert biological actions in distant organs and across multiple systems^4,5^. Conversely, the secreted form originates from alternative splicing, leading to the creation of a single domain (KL1). This form functions as an endocrine regulator of various cell surface glycoproteins^5^.

Scientific evidence indicates a decline in Klotho levels with age^6^, chronic stress^7^, and neurodegenerative diseases^8^. Interestingly, a decrease in Klotho expression has been linked to the onset of cognitive deficits^9^ and increased senescence in neural progenitor cells^10^. Conversely, Klotho overexpression seems to prolong the lifespan of animals^11^, enhance cognitive function, and improve learning and memory, regardless of age^12^. A recent study involving mice demonstrated that peripheral administration of Klotho enhanced cognitive performance^13^. Although the precise role of Klotho in the CNS is yet to be clearly defined, research has underscored its potential in boosting cognitive performance^13^.

The Insulin/Insulin-like Growth Factor 1(IGF-1) is a crucial pathway that influences aging and lifespan in animals^14,15^. In humans, insulin sensitivity is a hallmark of healthy longevity^16^. Moreover, soluble Klotho has a negative modulatory effect on IGF-1^17,18^, which results in a decrease in the activity of downstream signaling cascades, including the phosphoinositide 3-kinase (PI3K)/protein kinase B (PKB or AKT) pathways^17,19,20^.

The PI3-K/AKT signaling pathway plays a crucial role in integrating physiological responses associated with healthy aging^21^. When less active, it can contribute to lifespan extension^22^. The activation of PI3K, and consequently AKT, is triggered by the binding of insulin to the insulin receptor (IR), primarily through phosphorylation at Thr-308 induced by phosphoinositide-dependent kinase-1 (PDK-1). At this juncture, AKT can phosphorylate the mechanistic target of rapamycin (mTOR), a Ser/Thr kinase^23^. Full activation of AKT is achieved through its phosphorylation at Serine 473 by mTOR^21^. Once activated, AKT phosphorylates the Forkhead box O (FOXO) proteins, inhibiting FOXO nuclear translocation and its role as a transcriptional factor^24^. Thus, the Insulin/PI3K/AKT signaling pathway is instrumental in regulating members of the FOXO family.

Several studies have identified FoxO forkhead transcription factors (FOXOs) as significant regulators of longevity^25–27^. A potential mechanism is that, upon activation, FOXOs enhance the expression of catalase and manganese superoxide dismutase (MnSOD or SOD2), which detoxify harmful reactive oxygen species (ROS) derived from hydrogen peroxide and superoxide, respectively^28^. In the human retinal pigment epithelium, Klotho can reinstate the SOD2 gene expression levels under conditions of oxidative stress^29^. This finding aligns with a prior study that reported similar observations in HeLa cells^18^. Notably, the FOXO-3a member plays a pivotal role in oxidative stress resistance, autophagy, and apoptosis^30,31^.

Klotho has an important effect as an autophagy regulator in many types of cells owing to its influence on the IGF-1/PI3K/Akt/mTOR signaling pathway^32^. In addition, in hepatocarcinoma cells, the increase in exogenous klotho levels downregulates the phosphorylation levels of the IGF-1 receptor and the downstream AKT, ERK, and p70S6K proteins^33^. A similar effect was observed in a model of diabetic kidney disease, where Klotho gene deficiency enhanced the phosphorylation of the mammalian target of rapamycin (mTOR) and protein p70 ribosomal protein S6 kinase (p70S6K) in the kidney. However, when rat mesenchymal cells were incubated with exogenous Klotho prior to normal or high glucose 24 h exposure, Klotho inhibited mTOR phosphorylation stimulated by high glucose^34^. Therefore, we decided to verify whether Klotho was able to modulate these signaling pathways in neurons and astrocytes.

Furthermore, data from our group indicate that secreted Klotho (sKlotho) from neurons rapidly enhances aerobic glycolysis in astrocytes, which results in lactate release through the activation of fibroblast growth factor receptor 1 (FGFR1) and extracellular signal-regulated kinase 1/2 (ERK 1/2)^35^. Neurons directly use the lactate released by astrocytes to fulfill their energy demands during periods of high activity or indirectly by coupling the transamination of pyruvate to alanine with the conversion of glutamate to α-ketoglutarate. Lactate also plays a crucial role in lipid synthesis, which is essential for dendritic branching and synaptic plasticity^36,37^. Our recent findings also indicate that hypomorphic transgenic mice with the Klotho gene exhibit enhanced insulin signaling, resulting in increased AKT and mTOR activation and reduced FOXO activity in the hippocampus (unpublished data).

Mice deficient in Klotho exhibit extreme sensitivity to insulin and hypoglycemia. In contrast, transgenic mice overexpressing Klotho do not develop diabetes, despite showing moderate resistance to insulin and IGF-1^38^. Klotho mitigates insulin signaling in 3T3-L1 adipocytes, leading to a decrease in the phosphorylation of AKT, Glycogen Synthase Kinase-3 (GSK3β), and 6-phosphofructo-2-kinase/fructose-2,6-biphosphatase 3 (PFKFB3)^39^.

PFKFB3 serves as a regulator of cellular metabolism within the CNS. It controls the concentration of fructose-2,6-bisphosphate, a crucial activator of the phosphofructokinase-1 enzyme (PFK1) and glycolysis^40^. In neurons, PFKFB3 is ubiquitinated and degraded by the proteasome to maintain lower levels of fructose-2,6-bisphosphate and reduced PFK1 activity^41^. This process ensures that the pentose phosphate pathway (PPP) predominates in glucose metabolism, thereby elevating the levels of NADPH, which is instrumental in oxidative damage repair.

Our observations have led us to hypothesize that Klotho may activate FOXO-3a and enhance the expression of catalase and SOD2 in neurons and astrocytes, possibly owing to the inhibition of insulin/IGF-1 signaling. This process could facilitate the removal of reactive oxygen species and bolster cellular resistance to oxidative stress. Furthermore, we sought to determine whether Klotho could regulate PFKFB3 levels in the CNS as an additional cellular protective strategy. Our findings indeed suggest that Klotho functions as a potent antioxidant in astrocytes, augmenting FOXO-3a activity and catalase expression, thereby protecting them from death. However, Klotho at 1 nM was unable to prevent neuronal death following severe oxidative insult, but it did reduce PFKFB3 levels, thereby enhancing proteasome activity.

## Methods

### Chemicals and kits

Cell culture reagents were purchased from Thermo Fisher Scientific (Waltham, MA, U.S.A.). The protein assay kit, obtained from Bio-Rad (Hercules, CA, U.S.A.) was utilized in accordance with the manufacturer´s instructions. Routine reagents were purchased from Sigma Chemicals (St. Louis, MO, U.S.A.), while recombinant α-Klotho was acquired from R&D Systems (1819-KL-050; Minnesota, MN, U.S.A). All solutions were freshly prepared prior to use.

### Animals and ethics

All procedures adhered to the ARRIVE guidelines (https://arriveguidelines.org), which are grounded in the Ethical Principle in Animal Research endorsed by the Brazilian College of Animal Experimentation (CONCEA). These procedures received approval from the Ethical Committee for Animal Research (CEUA) at the Biomedical Sciences Institute of the University of São Paulo, São Paulo, São Paulo State, Brazil. The protocol was officially registered under the number 12/2016 CEUA for animals utilized in experimentation.

#### Primary culture of cortical glial cells

Primary cortical glial cell cultures were derived from postnatal (P1-P3) mice of both sexes^42^. The meninges were carefully removed, and the cortices were dissected in cold Hank’s Balanced Salt Solution (HBSS). The tissue was then dissociated using 0.25% trypsin at 37 °C for 5 min, after which Dulbecco's Modified Eagle Medium (DMEM) (supplemented with 4 mM glutamine, 10% Hyclone FetalOne III serum [GE Healthcare, USA], and 1% penicillin/streptomycin) was added. The cells were further dissociated using a Pasteur pipette, filtered, and plated at a density of 1.5 × 10^4^ cells/cm^2^ in a T75 flask. The medium was refreshed every 3 days. The cultures were incubated until they achieved confluency, typically within 10–14 days. The cells were then digested with 0.25% trypsin at 37 °C for 10 min to facilitate detachment from the flask. Digestion was halted by adding DMEM with 10% fetal bovine serum (FBS), after which the cells were centrifuged for 2 min at 2000 rpm. The resulting pellet was resuspended in DMEM 10% FBS and counted for plating. Each well in a 6-well plate received 1 × 10^6^ cells. For a 24-well plate, cells were seeded at a density of 1 × 10^4^ per well. The flasks were then incubated in an orbital shaker at 37 °C and 180 r/min for 15 h, following a previously described method to separate astrocytes from other glial cells^35^. The resulting culture, used for the experiments, was deemed an astroglial-enriched culture, as it contained 91.2% glial fibrillary acidic protein-labeled cells. Representative photomicrographs of the culture phenotype are provided in the supplementary material (see Supplementary Fig. S1 online).

#### Primary mouse (embryonic) cortical neuronal culture

Primary cortical neuronal cultures were prepared from both male and female C57BL/6 mouse embryos on gestational days 16th–17th (E16-17). The meninges were excised from the brain, and the cortices were sectioned into small fragments and incubated in a 2 mg/mL trypsin solution for 20 min at 37 °C in a 5% CO_2_ incubator. Following the removal of the trypsin solution, the tissue was rinsed twice with HBSS. The tissue was then dissociated in HBSS containing 0.1 mg/mL DNAse through mechanical trituration using a glass pipette. The cells were counted and seeded at a density of 1 × 10^5^ cells/cm^2^ on dishes pre-coated with polyethyleneimine (Sigma-Aldrich). The neurons were maintained for 2 weeks in Neurobasal medium (GIBCO) supplemented with B27 (GIBCO), 2 mM L-glutamine, 100 U/mL penicillin, 100 mg/mL streptomycin, and 0.25 mg/mL amphotericin B. A representative photomicrograph of the culture phenotype is presented in Supplementary Fig. S2, available in the supplementary material. On average, the primary culture comprised 80% neurons.

##### Transfection with FOXO reporter plasmid

The activity of the FOXO transcription factor was assessed using the pGL-3 × DBE plasmid (Promega)^43^, which incorporates three iterations of FOXO-responsive promoters and encodes firefly luciferase. Concurrently, the pRL-TK plasmid (Promega), expressing renilla luciferase constitutively, was transfected as an internal control. Astrocytes were seeded in 60 mm diameter plates (1 × 10^6^ cells/plate) and transfected after 48 h (approximately 60% confluence) using Fugene 6 (Promega) with 1 µg DBE DNA, 0.2 µg pRL-TK DNA, and a DNA: Fugene ratio of 1:6. Following 48 h of transfection, the cells were treated by washing twice with phosphate buffered saline (PBS) and incubating for 24 h in DMEM containing either 4.5 g/L or 1 g/L glucose concentration, with or without 1 nM Klotho. Post-treatment, the cells were lysed as per the Dual-Luciferase^®^ Reporter Assay system (Promega) guidelines. Equal sample volumes were used, and the luminescence was developed and read according to the manufacturer's instructions (Promega) on opaque white plates using a Synergy H1 Hybrid Multi-Mode plate reader (BioTek). The results are presented as the ratio of firefly luciferase fluorescence to renilla luciferase, normalized to the mean of the control group.

##### Cell viability assay: color formazan reduction (MTT)

The MTT assays were conducted as previously described^44,45^. This method relies on the ability of viable cells to convert tetrazolium salt (MTT) into formazan, a colored compound. Cells were pretreated with either PBS or Klotho for a duration of 24 h, followed by a 30-min exposure to H_2_O_2_. Cell viability was ascertained by incubating cells with MTT (12 mM) in the cell culture medium for 2 h at 37 °C, 24 h post the final treatment. The dark crystals that formed were solubilized with DMSO, and the absorbance was measured at a wavelength of 570 nm using a microplate reader. The MTT (%) was calculated using the formula: (absorbance of the sample − absorbance of DMSO)/(absorbance of the control − absorbance of DMSO) × 100.

##### Western blotting

Total protein extracts (nuclear and cytoplasmic) were obtained by collecting and pelleting cells, which were then homogenized in RIPA lysis buffer containing protease and phosphatase inhibitors (20 mM Tris–HCl, 150 mM NaCl, 1 mM Na_2_EDTA, 1% NP-40, 1% sodium deoxycholate, 2.5 mM sodium pyrophosphate, 1 mM β-glycerophosphate, 1 mM sodium orthovanadate, and mini protease inhibitor cocktail). The Bradford method was used to determine protein concentration. Equal protein amounts (15 µg) were separated on 4–12% bis–tris NuPAGE gels (Invitrogen) and transferred to a nitrocellulose membrane. The membrane was then incubated with a 5% bovine serum albumin (BSA) solution in Tris-buffered saline with tween (TBS-T) (10 mM Tris, pH 8.0, 150 mM NaCl, 0.5% Tween 20) for 2 h. The membranes were incubated overnight at 4 °C in primary antibody solution (TBST, 1% BSA) with pAKT Ser473 (1:1000, Cell Signaling #4056), AKT pan (1:1000, Cell Signaling #4685), p-mTOR Ser2448 (1:1000, Cell Signaling #5536), mTOR (1:1000, Cell Signaling #2983), FOXO3 (1:1000, Cell Signaling #2497), p-FOXO3 SER253 (1:1000, Cell Signaling #9466), Catalase (1:1000, Santa Cruz, #Sc50508), PFKFB3 (1:1000, Cell Signaling #13123), and Beta-actin (1:1500, Sigma Chemical #A2066). After three washes with TBS-T, the membranes were incubated for 2 h with a secondary antibody solution (TBS-T, 1% BSA) containing horseradish peroxidase-conjugated antibodies anti-rabbit or anti-mouse IgG-Fc (1:4000). Following three additional washes with TBS-T, proteins recognized by the antibodies were revealed using an ECL system (Pierce ECL Western Blotting Substrate, Thermo Scientific). The photo documentation system DP-001-FDC (VilberLourmat, Torcy, France) and N.I.H. ImageJ software (http://rsb.info.nih.gov/ij) were used to standardize and quantify the immunoblots. Multiple exposure times were analyzed to ensure the linearity of the band intensities.

##### Proteasome activity assay

The proteasome activity was measured using the Proteasome 20S Activity Assay kit (Sigma-Aldrich). Neurons were plated into a 96-well black microplate with a flat and translucent bottom. Cells were cultured under normal conditions for 14 days and then subjected to different conditions: (i) 24 h with complete medium (control group); (ii) 24 h with medium without insulin, supplemented with B27 minus insulin (Gibco, Catalog number: A1895601); (iii) 24 h with 1 nM of Klotho, and (iv) with the proteasome inhibitor MG132 at 50 µM for 2 h before performing the assay. The assay relies on the proteosome complex performing the proteolytic breakdown of the fluorogenic substrate, LLVY-R110. Cleavage of LLVY-R110 by the proteasome generates the R110 product, which shows intense green fluorescence (λex = 480–500 nm and λem = 520–530 nm). Following treatments, the culture medium was immediately replaced with an LLVY-R110 solution, and incubation lasted for 1 h. Fluorescence reading was conducted using a Synergy H1 Hybrid Multi-Mode plate reader (BioTek). Fluorescence values were corrected for the blank (cell-free well) and are presented as a function of the mean values of the control group. Three independent neuronal cultures were analyzed, and the final value expressed in the resulting graph represents an average of 24 wells per group/ per culture. The control group had 22 wells in each plate.

##### Immunoprecipitation

To assess protein phosphorylation and ubiquitination levels, astrocyte and neuron samples were examined through immunoprecipitation. These samples were obtained from pools of six wells from six-well plates, ensuring sufficient protein content (an average of 6 × 10^6^ cells). The cells were gathered and homogenized in non-denaturing lysis buffer (20 mM Tris–HCl, 137 mM NaCl, 10% glycerol, 1% NP-40, and 2 mM EDTA, supplemented with 30 mM NaF phosphatase inhibitors, 20 mM sodium pyrophosphate, 5 mM β-glycerophosphate, 2 µg/ml leupeptin protease inhibitors, and 2 µg/mL antipain) using ultrasonication on ice. A total of 100 µg of protein (diluted in 400 µL of buffer) was incubated with 2 µg of antibody against phosphotyrosine (clone 4g10, Millipore) or against mono- and polyubiquitin K^29^, K^48^, K^63^ (clone FK2, Enzo Life Sciences). For their respective negative controls, 2 µg of rabbit IgG or mouse IgG were used. The samples were incubated overnight with light orbital shaking. Subsequently, 20 µL of the suspension containing 50% agarose microspheres with protein A/G covalently immobilized was added, followed by incubation under light agitation for 3 h. The samples were then centrifuged and washed five times with ice-cold PBS. The final precipitate was resuspended in 20 µL of three-fold concentrated SDS sample buffer, homogenized, and centrifuged again, followed by heating at 70 °C for 10 min. After an additional centrifugation, the samples were loaded onto an SDS PAGE gel and analyzed by western blotting against PFKB3 (1:1000, Abcam #Ab96699) for FK2 and PKM2 (1:1000, Cell Signaling #3198). This assay was conducted on three independent cell cultures.

### Ethical approval and consent to participate

The animals used, and the procedures performed were approved by the Ethics Committee for the Use of Animals (CEUA) of the Institute of Biomedical Sciences, protocol registered under number 63 on page 07 of book 03, approved on May 29, 2013, and extended on May 12, 2017 (Of. CEUA. 014.2017) until May 29, 2021. The use of animals and all experimental procedures were deemed appropriate and in accordance with the Ethical Principles of Animal Experimentation adopted by the Brazilian Society of Laboratory Animal Science (SBCAL).

## Results

### Klotho decreases AKT and mTOR phosphorylation in neurons

In a prior study conducted by our group, we observed that treating astrocytes with 1 nM Klotho led to a rapid increase in aerobic glycolysis and lactate release via ERK activation, coupled with a simultaneous reduction in AKT phosphorylation^35^. Consequently, to ascertain whether 1 nM Klotho could modulate the Insulin/AKT/mTOR and FOXO3a signaling pathways in neurons, we treated primary cortical neurons with 1 nM Klotho for 24 h and subsequently analyzed them using western blotting. For the purpose of a positive control, cells were exposed to 100 nM insulin for the same duration. This assay involved the treatment and analysis of four independent cultures.

**Figure 1:**
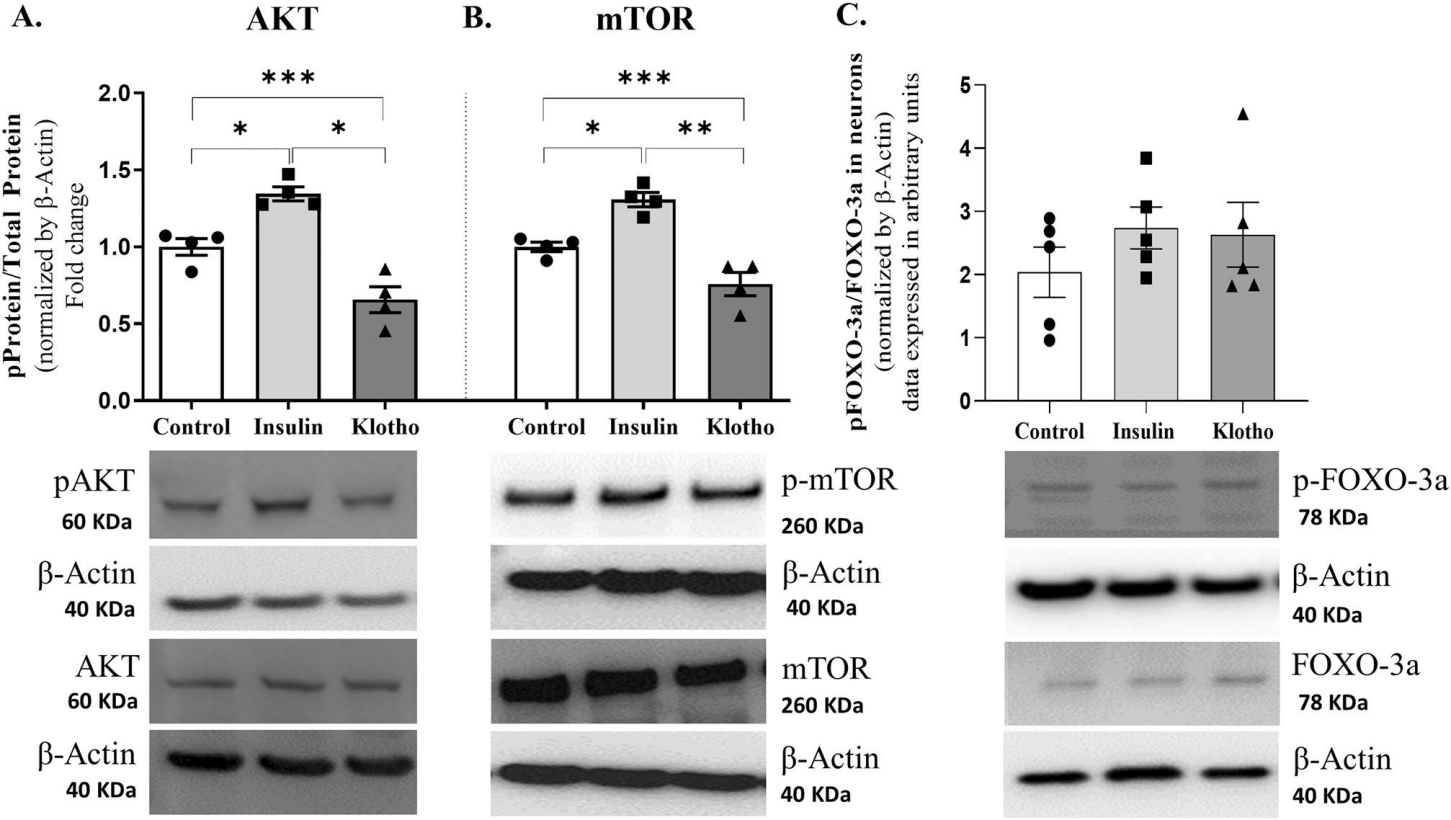
Phosphorylation levels of AKT, mTOR, and FOXO3a after 24 h of treatment with insulin 100 nM or Klotho 1 nM in primary neuronal culture. (**A**) Statistical analysis indicated that insulin increases AKT phosphorylation on Ser473, while Klotho decreased AKT phosphorylation. One-way ANOVA followed by Tukey’s multiple comparisons tests; pAKT/AKT [F = 29.05; *P* = 0.0001; R squared: 0.8659]. (**B**) Statistical analysis of mTOR phosphorylation levels. One-way ANOVA followed by Tukey’s multiple comparisons tests indicated that insulin increased phosphor-mTOR, as expected, whereas Klotho decreased phospho-mTOR[F = 25.78; *P* = 0.0002; R squared = 0.8514]. (**C**) Statistical analysis of FOXO-3a phosphorylation levels. One-way ANOVA indicated the absence of significant difference [F = 0.8042; R squared = 0.1182, *P* = 0.4702]. Below the graphs are the representative bands of each group in western blot autoradiographs. The original autoradiographs are available in Supplementary Fig. S3. N = 4–5 independent cell cultures. *P < 0.05 and ***P = 0.0001.

As anticipated, insulin treatment increased AKT phosphorylation at Ser473 (Fig. 1A) and mTOR phosphorylation at Ser2448 (Fig. 1B). In contrast, Klotho treatment induced a reverse response, diminishing AKT and mTOR phosphorylation (p-AKT/AKT: control = 1.00 ± 0.05487; insulin = 1.346 ± 0.046; Klotho = 0.6568 ± 0.08439; p-mTOR/mTOR: control = 1.00 ± 0.03109; insulin = 1.308 ± 0.7588; Klotho = 0.7588 ± 0.0756; n = 4). The subsequent step involved verifying whether Klotho modulates FOXO3a phosphorylation. A western blot assay indicated that after a 24-h treatment period, no significant difference was detected in S253 phosphorylation levels (Fig. 1C). Each sample used in the western blot analysis contained a minimum of 2 × 10^6^ cells. Each dot represents an independent cell culture.

### Klotho promotes antioxidant defense through FOXO3a activity in astrocytes

Following 24 h of treatment with either 1 nM Klotho or 100 nM insulin, FOXO3a phosphorylation levels in astrocytes were assessed using western blot analysis. Lysates were obtained from either a single well or two wells of a 6-well plate, yielding a total of 2 × 10^6^ cells. Each data point in the graphs corresponds to a lysate derived from an independent cell culture.

**Figure 2:**
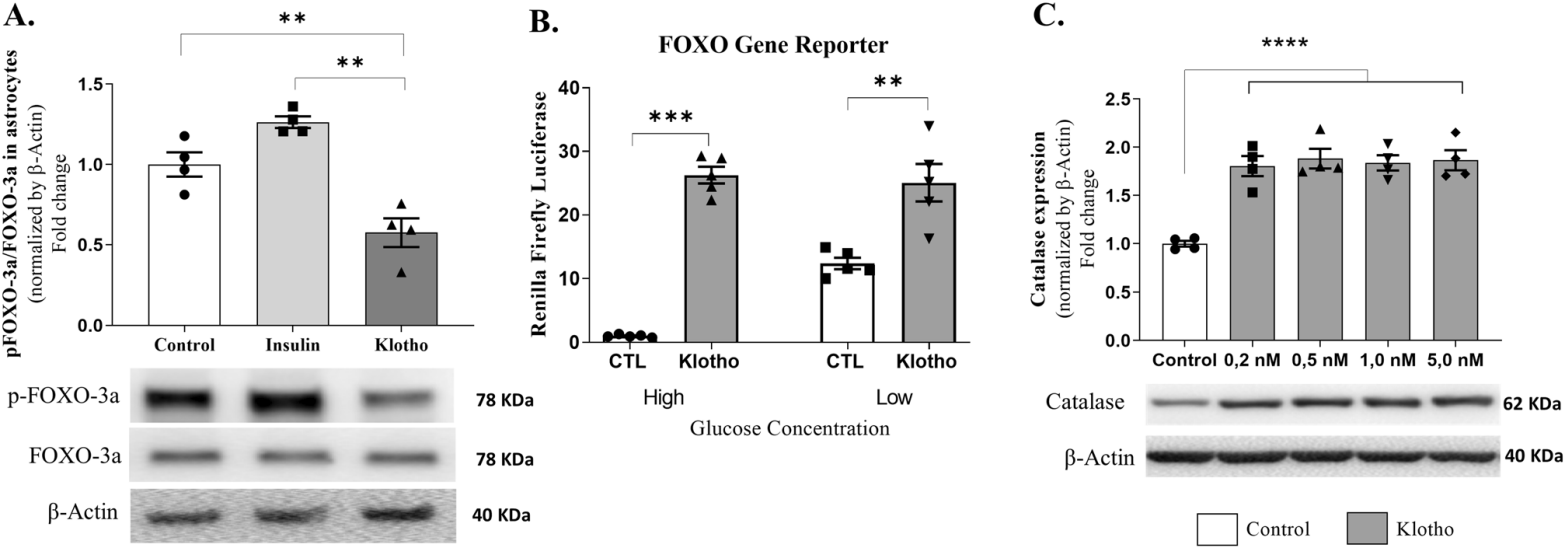
Klotho affects FOXO-3a activity and catalase activity in astrocytes. (**A**) Western blot assay was performed to measure phosphorylation levels of FOXO-3a. Statistical analysis suggested that Klotho treatment decreased the FOXO-3a inhibitory phosphorylation. One way ANOVA followed by Tukey's multiple comparisons tests; [F = 23.86; *P* = 0.0003; R squared = 0.8413]. Below the graph is the representative western blot digital images of p-FOXO-3a, FOXO-3a, and β-actin. (**B**) FOXO gene reporter activity. After 48 h of plasmid transfections, treatments were performed by incubating cells for 24 h with DMEM containing high (4.5 g/L) or low (1 g/L) concentrations of glucose, with or without 1 nM Klotho. Results represent the ratio of fluorescence of firefly luciferase to renilla luciferase, normalized to the mean of the control group (DMEM high glucose). One-way ANOVA statistical analysis followed by Tukey’s multiple comparison tests. FOXO-3a activity was increased in all groups: CTL High vs. CTL Low (mean difference: − 11.35; q = 6.782), CTL High versus Klotho High (mean difference: − 25.26; q = 15.09), CTL High versus Klotho Low (mean difference: − 24.06; q = 14.38), CTL Low versus Klotho High (mean difference: − 13.91; q = 8.313), and CTL Low versus Klotho Low (mean difference: − 12.71; q = 7.598). No difference was observed between Klotho high and Klotho low glucose (mean difference: 1.196; q: 0.7148). *P* < 0.001, F = 50.66, and R squared = 0.9048. (**C**) Catalase relative activity. Statistical analysis suggested that all klotho concentrations increased catalase expression compared to that of the control group. [One-way ANOVA followed by Tukey’s multiple comparison test. F = 18.38; R squared 0.8386; *P* < 0.0001]. *P < 0.05; ***P < 0.0001. The original autoradiographs are available in Supplementary Figs. S4 and S5. Lysates were obtained from two or three wells of a 6-well plate to reach 2 × 10^6^ cells. Each point in the graphs represents a lysate obtained from independent cell cultures, and four independent cell cultures were analyzed in these assays.

Our findings indicate that the application of Klotho prevented the inhibitory phosphorylation of the FOXO3a transcription factor in astrocytes, as depicted in Fig. 2A. Conversely, insulin demonstrated its anticipated inhibitory impact on this protein (control = 1.00 ± 0.07624; insulin = 1.26 ± 0.03678; Klotho = 0.58 ± 0.08931; n = 4).

To evaluate the transcriptional activity of the FOXO transcription factor directly, astrocytes were transfected with a plasmid housing copies of (i) the FOXO-responsive sequence (pGL-3xDBE), serving as the promoter unit of a gene encoding firefly luciferase and (ii) plasmids carrying a gene encoding promoter-regulated renilla luciferase constitutive thymidine kinase (pRL-TK), which served as an internal control for the transfection rate. Following transfection, astrocytes were maintained for 24 h under varying culture conditions (in media with high or low glucose concentrations), with or without 1 nM Klotho. The relative luminescence was then measured to determine the transcription rate of the luciferase gene and, by extension, the activity of the FOXO transcription factor. The results indicated that a lower glucose concentration stimulated FOXO activity. Interestingly, Klotho augmented transcription factor activity in both high and low glucose concentrations, implying a potential role for Klotho in modulating FOXO activity in astrocytes (high glucose control = 1.00 ± 0.09658; low glucose control = 12.35 ± 0.8978; high glucose klotho = 26.26 ± 1.316; low glucose klotho = 25.06 ± 2.942; n = 5) (Fig. 2B).

The influence of FOXO on genes associated with antioxidant defense, such as manganese superoxide dismutase (MnSOD or SOD2) and catalase, has been previously described^46^. To explore the capacity of Klotho to stimulate catalase expression in astrocytes, a western blot analysis was conducted following a 24-h treatment period with varying concentrations of Klotho. The analysis indicated that catalase expression was induced by Klotho treatment across all tested concentrations, demonstrating a dose-independent response (Fig. 2C). This evidence implies that Klotho may activate antioxidant defense mechanisms within astrocytes, potentially via its regulation of FOXO transcriptional activity (control = 1.00 ± 0.02975; Klotho 0.2 nM = 1.80 ± 0.1034; 0.5 nM = 1.88 ± 0.103; 1.0 nM = 1.84 ± 0.07988; 5.0 nM = 1.87 ± 0.1039; n = 4).

### Klotho protects astrocytes but not neurons against oxidative insult

**Figure 3:**
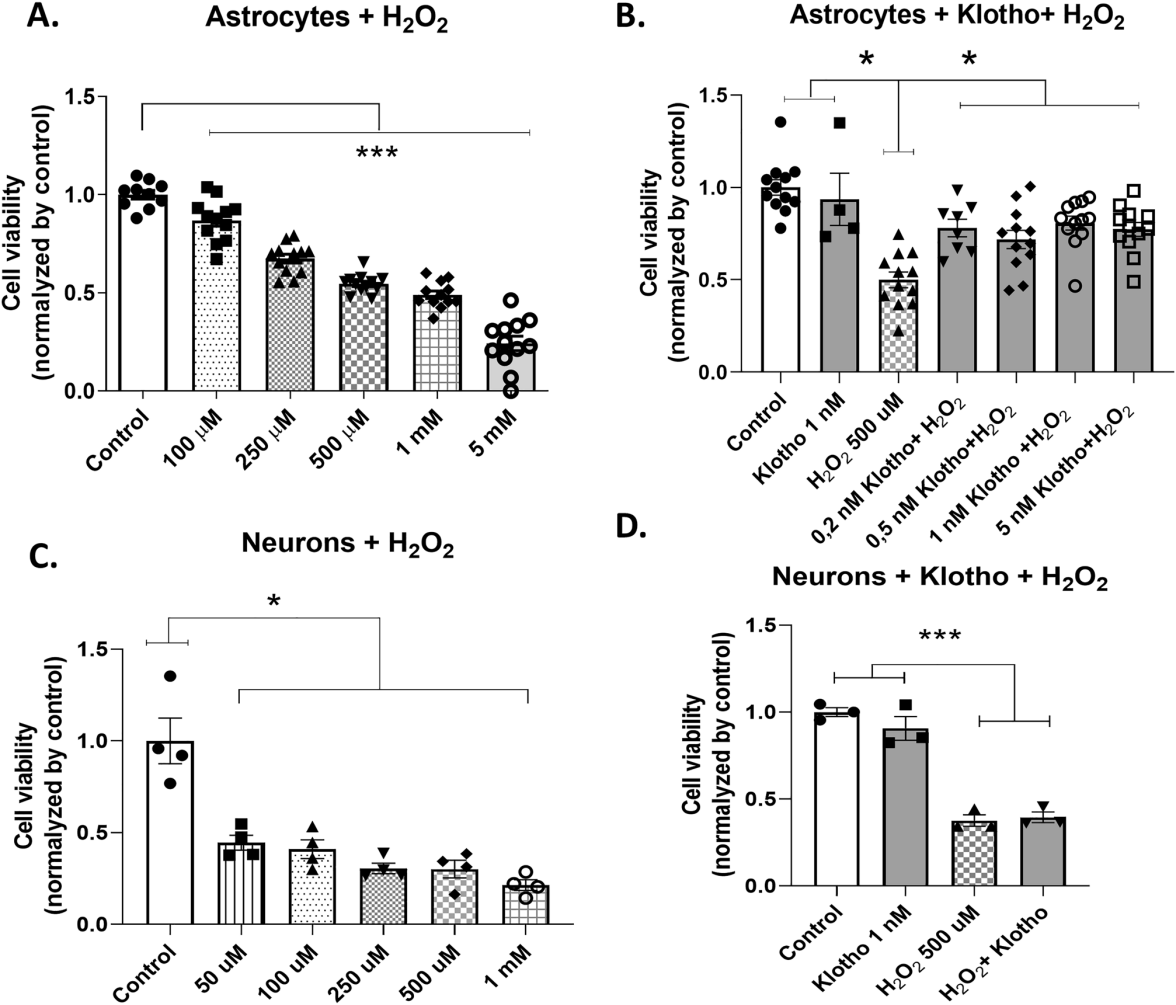
Viability of astrocytes and neurons after oxidative insult by different concentrations of H_2_O_2_ and the protective effects of Klotho. Neurons and astrocytes were pretreated with PBS or Klotho with different concentrations. After 24 h, the cells were exposed to a 30-min insult with H_2_O_2_ at different concentrations. Cellular viability was measured by MTT. (**A**) Astrocytes were challenged with different concentrations of H_2_O_2_. All concentrations tested were sufficient to reduce their viability compared to the control group. One-way ANOVA followed by Tukey’s multiple comparisons tests [F = 110.5; R squared = 0.8962; *P* < 0.0001, n = 12]. A concentration of 500 µM was selected. (**B**) Astrocytes were pretreated with different concentrations of Klotho and challenged with 500 µM H_2_O_2_. Results suggest that 1 nM Klotho alone was not able to alter cell viability, although 500 µM H_2_O_2_ decreased cell viability, as expected. The pretreatment with all different Klotho concentrations protected astrocytes from death. One-way ANOVA followed by Tukey’s multiple comparisons tests [F = 11.83; R squared = 0.5221; *P* < 0.0001, n = 4–12]. (**C**) Neurons were challenged with different concentrations of H_2_O_2_. All concentrations tested were sufficient to reduce their viability compared to the control group. One-way ANOVA followed by Tukey's multiple comparisons tests [F = 20.36; R squared = 0.8498; *P* < 0.0001, n = 4]. (**d**) Neurons were pretreated with 1 nM Klotho for 24 h and challenged with 500 µM H_2_O_2_ for 30 min. Compared to the control group, as expected, H_2_O_2_ altered neuronal viability but Klotho did not. However, pretreatment with Klotho did not avoid cell death caused by oxidative insult. Therefore, compared to the control group, both groups (H_2_O_2_ and H_2_O_2_ + Klotho) had a decreased viability. One-way ANOVA followed by Tukey’s multiple comparisons tests [F = 59.01; R squared = 0.9568; *P* < 0.0001, n = 3]. *P < 0.05; ***P < 0.0001.

We conducted an investigation into the potential protective role of Klotho against oxidative damage. The viability of astrocytes and neurons exposed to varying concentrations of H_2_O_2_ was assessed using the MTT assay. Our results indicated a significant reduction in astrocyte viability across all tested H_2_O_2_ concentrations (Fig. 3A). To further evaluate the protective effect of Klotho against H_2_O_2_-induced damage, we selected a concentration that represented an intermediate level of insult, resulting in slightly less than 50% cell death. For this purpose, we opted for a concentration of 500 µM H_2_O_2._ Astrocytes were pretreated with either recombinant Klotho or a vehicle (PBS) at varying concentrations for 24 h prior to a 30-min exposure to H_2_O_2_. Our findings demonstrated that all tested Klotho concentrations effectively mitigated astrocyte death induced by H_2_O_2_ (Fig. 3B). This suggests that Klotho may play a protective role against intermediate oxidative damage in astrocytes (control = 1.00 ± 0.04143; Klotho 5 nM = 0.9354 ± 0.1413; H_2_O_2_ 500 µM = 0.4999 ± 0.04248; H_2_O_2_ + Klotho 0.2 nM = 0.7802 ± 0.04736; H_2_O_2_ + Klotho 0.5 nM = 0.7189 ± 0.04975; H_2_O_2_ + Klotho 1.0 nM = 0.8071 ± 0. 03,735; H_2_O_2_ + 5.0 nM Klotho = 0.7732 ± 0.03756; n = 4–6) (Fig. 3B).

All tested concentrations of H_2_O_2_ significantly decreased cell viability in neurons compared to the control group. In neuronal culture, the same H_2_O_2_ concentration that was used in astrocytes constituted a severe oxidative insult, killing nearly 75% of the neurons (Fig. 3C). Our lab previously demonstrated that 1 nM Klotho for 24 h could protect neurons from death following exposure to LPS-activated microglia-conditioned medium^47^. Consequently, we sought to determine if the same concentration could protect neurons from a severe oxidative insult. However, pretreatment with 1 nM Klotho did not offer neuroprotection against cell death induced by 30 min of exposure to 500 µM H_2_O_2_ (Fig. 3D).

### Klotho induces PFKFB3 ubiquitination and proteasome activity in neurons

**Figure 4:**
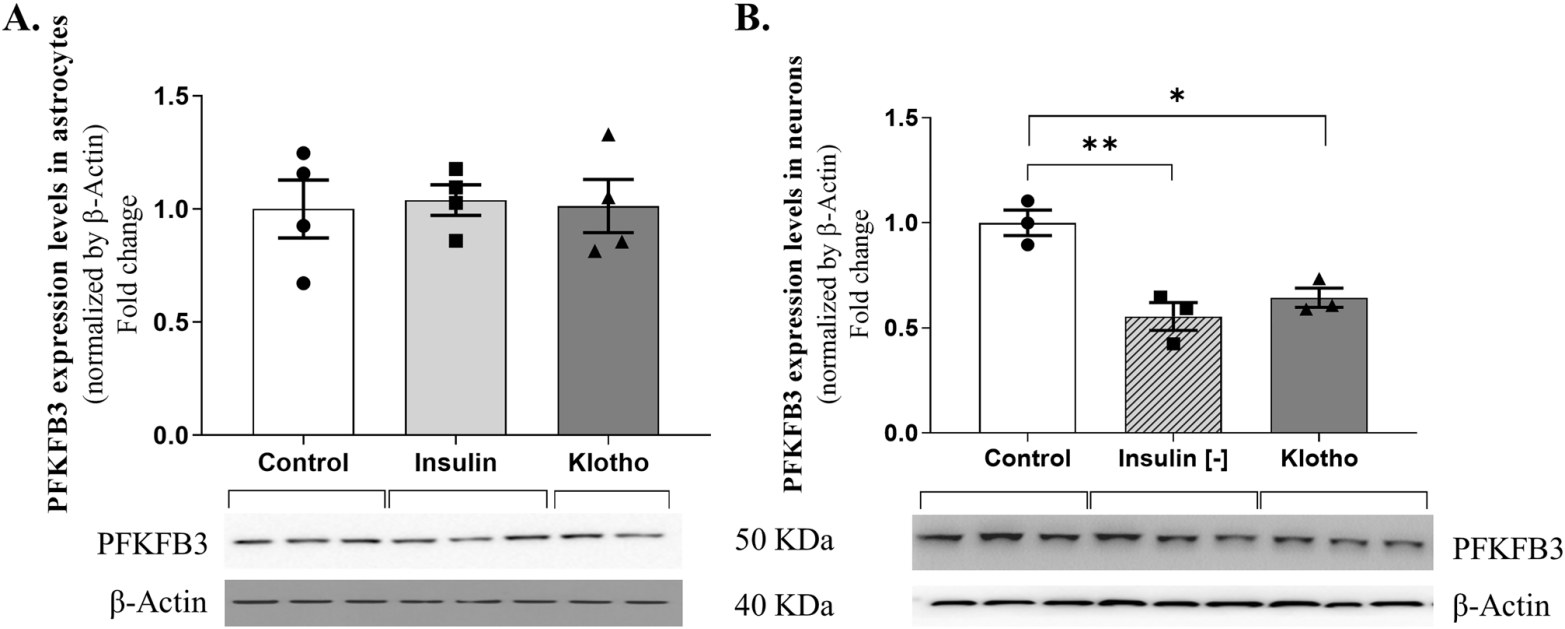
PFKFB3 levels measured by western blot in neurons and astrocytes, normalized by the control. Primary cultures of astrocytes and neurons were treated with 1 nM Klotho for 24 h in the presence or absence of insulin. (**A**) In astrocytes, the level of PFKFB3 was not altered by insulin or Klotho treatment within 24 h. (**B**) Tukey’s multiple comparisons test indicated that in neurons, the cells that were deprived of insulin had reduced PFKFB3 levels compared to those of the control group [mean difference 0.4455, q = 7.641, *P* = 0.004]. Similarly, the cell treated with Klotho had reduced PFKFB3 levels compared to the those of the control group [mean difference: 0.3563, q = 6.111, *P* = 0.0118]. One-way ANOVA [F = 16.35; R squared = 0.8449; *P* = 0.0037]. Below the graphs are representative images of a western blot for PFKFB3 and β-actin. N = 3–4 independent cultures. The original autoradiographs are available in Supplementary Fig. S6.

The literature has previously indicated that Klotho suppresses PFKFB3 activity in adipocytes^39^. Maintaining low PFKFB3 levels in neurons could potentially facilitate the PPP and augment NADPH levels, which are crucial for oxidative damage repair. To explore the potential role of Klotho in modulating PFKFB3 expression, primary astrocyte cultures and hippocampal cortical neurons were exposed to a 24-h treatment with 1 nM Klotho. Subsequently, PFKFB3 levels were evaluated using western blotting (Fig. 4). All original blots can be found in Supplementary Fig. S6.

Treatment with either 1 nM Klotho or 100 nM insulin did not alter PFKFB3 levels in astrocytes. However, a 24-h absence of insulin or treatment with Klotho resulted in a decrease in PFKFB3 levels in neurons (Fig. 4B) [Neurons: control = 1.00 ± 0.06043; insulin [−] = 0.5545 ± 0.06674; Klotho = 0.6437 ± 0.04574; n = 3; Astrocytes: control = 1.00 ± 0.1287; insulin [+] = 1.04 ± 0.06751; Klotho = 1.013 ± 0.1175; n = 4].

Based on the transcriptome results from a neuronal microarray^35^, we decided to assess both the proteasome activity and the levels of poly-ubiquitinated proteins in neurons. These neurons were either treated with 1 nM Klotho for 24 h or maintained without insulin for the same duration. To measure proteasomal activity, we included a negative control group, which consisted of cells treated with MG 132 (50 µM) for 2 h. Each dot on the graph represents the average of values measured in 22–24 wells of a 96-well plate from a single cell culture. We conducted this analysis of proteasome activity across three independent cell cultures.

**Figure 5:**
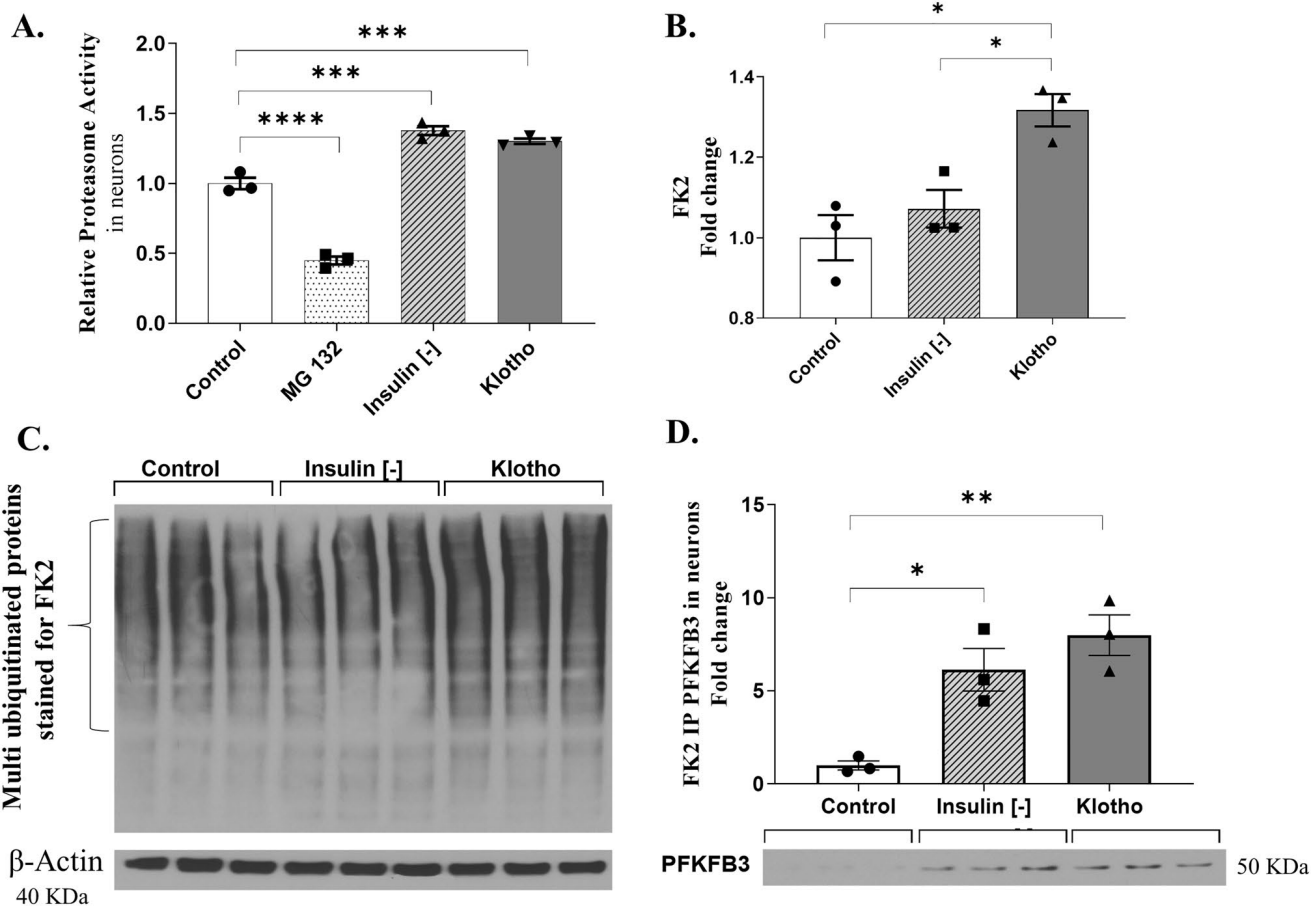
Relative proteasome activity and levels of ubiquitination measured by immunoprecipitation in neurons. (**A**) For the measurement of proteasome activity, four groups were analyzed: the control group, a positive control group whose cells received a proteasome inhibitor (MG 132, 50 µM, for 2 h), the insulin-deprived group, and the group treated with Klotho. One-way ANOVA suggested a difference among groups [F = 180.5; R squared = 0.9854; *P* < 0.0001]. The post-test Tukey indicated that the group treated with the proteasome inhibitor MG132 had decreased proteasome activity compared to all the other groups [control vs. MG132: mean diff.: 0.5502; q = 17.55; *P* < 0.0001. MG132 vs. Insulin (−): mean diff: − 0.4498; q = 29.60; *P* < 0.0001. MG132 vs. Klotho: mean diff.: − 0.4498; q = 27.18, *P* < 0.0001]. Insulin deprivation increased proteasome activity compared to MG132 and control [insulin (−) vs. control: mean diff. − 0.3774; q = 12.04; *P* = 0.0001]. Klotho treatment increased proteasome activity compared to MG132 and control [Klotho vs. control: Mean diff: − 0.3016; q = 9.623, *P* = 0.0006]. N = 3 independent cellular cultures. Each dot in the graph represents an average of values measured in 22–24 wells of a 96-well plate from a single cell culture. (**B**) Western blot for antibody against polyubiquitin (FK2) proteins. Klotho treatment increases polyubiquitination compared to the control and insulin deprivation groups. One-way ANOVA followed by Tukey’s multiple comparisons tests [F = 11.91; R squared 0.7988; *P* = 0.0081]; N = 3 independent neuron culture. (**C**) Representative membrane of FK2 immunostaining. (**D**) Immunoprecipitation assay against clone FK2 followed by western blot against PFKFB3. Representative images of the western blot are below the graph. Results suggest that either insulin deprivation or Klotho treatment enabled increase in the PFKFB3 ubiquitination in neurons. One-way ANOVA [F = 15.39; R squared = 0.8369; *P* = 0.0043] followed by Tukey’s multiple comparisons tests [Insulin (-) vs. control: mean diff: − 5.137, q = 5.563, *P* = 0.0180] and [Klotho vs. control: mean diff: − 6.993, q = 7.573, *P* = 0.0042], N = 3 independent assays. Samples were obtained from pools of a 6-well plate (an average of 6 × 10^6^ cells). The original autoradiographs are available in Supplementary Fig. S7.

Figure 5A demonstrates that proteasome activity was effectively suppressed by MG 132 treatment. Intriguingly, both the absence of insulin and treatment with recombinant Klotho were observed to enhance the proteolytic activity of the proteasome complex (control = 1.00 ± 0.04129; MG 132 = 0.4498 ± 0.02822; insulin [−] = 1.377 ± 0.03228; Klotho = 1.302 ± 0.01965; n = 3). In a similar vein, Klotho treatment resulted in an increase in the content of polyubiquitinated proteins, an effect that was not observed in cells that had been maintained without insulin for 24 h (control = 1.00 ± 0.05619; insulin [−] = 1.072 ± 0.04676; Klotho = 1.137 ± 0.04026; n = 3) (Fig. 5b).

To verify whether the observed decrease in PFKFB3 in neurons was a result of Klotho’s modulatory action on ubiquitination, we assessed the levels of polyubiquitinated PFKFB3 through immunoprecipitation. We employed an antibody against polyubiquitin (FK2) (Fig. 5c), followed by western blotting against PFKFB3 (Fig. 5D). The findings suggest that the absence of insulin and the administration of Klotho treatment induced PFKFB3 ubiquitination in neurons (control = 1.00 ± 0.245; insulin [−] = 6.14 ± 1.143; Klotho = 8.00 ± 1.092; n = 3).

## Discussion

The brain requires substantial amounts of oxygen and glucose to sustain high levels of neuronal activity^48^. Neurons possess a limited capacity to metabolize energy substrates other than glucose, which accounts for the abundance of mitochondria and the intense oxidative phosphorylation observed^49^. Given that optimal neuronal function and, consequently therefore, healthy cognitive performance necessitate proper metabolic cooperation between astrocytes and neurons, this study aimed to determine whether exogenous Klotho could inhibit insulin/IGF-1 signaling in neurons and activate FOXO-3a. As previously noted, FOXOs enhance the expression of catalase, which detoxifies ROS from hydrogen peroxide. Additionally, we aimed to investigate whether Klotho could modulate PFKFB3 levels in the CNS. PFKFB3, a crucial regulator of cellular metabolism^40^, should be maintained at low levels in neurons to improve antioxidant defenses.

The beneficial impact of Klotho on the activity of FOXO transcription factors is well-documented^18,50^. Klotho can mitigate the suppressive effect of AKT on FOXO transcription factors by negatively modulating the PI3K/AKT pathway. This allows the transcription factors to relocate to the nucleus and become active. Consistent with our previous findings from astrocytes, Klotho has also been observed to reduce AKT and mTOR phosphorylation levels in cortical neurons (Fig. 1A), which is in agreement with our previous results from astrocytes^35^.

The subsequent step involved verifying the effects of Klotho on FOXO3a activity. Initially, we assessed its phosphorylation levels following a 24-h treatment with either 1 nM Klotho or 100 nM insulin in neurons. However, no discernible difference was noted in the FOXO3a phosphorylation levels within neurons (Fig. 1B). In contrast, within astrocytes, Klotho treatment impeded the inhibitory phosphorylation of the FOXO3a transcription factor. As anticipated, insulin demonstrated its inhibitory effect on this protein (Fig. 2A). This disparity in phosphorylation patterns suggests that FOXO3a activity in neurons and astrocytes is subject to distinct regulatory mechanisms. Indeed, Du et al. have demonstrated that insulin treatment does not impact the subcellular localization of FOXO3a^51^. Unlike astrocytes, FOXO3a activity in neurons responded to DNA damage or astrogliosis^52^. Interestingly, a region-specific pattern of FOXO3a regulation was observed, with a decrease in FOXO3a activity in the cortex, but not in the hippocampus of aged mice^50^. Conversely, another study found that when primary hippocampal neurons were treated with 4 µg/mL Klotho for 24 h, there was a sustained increase in AKT activation and inhibitory FOXO3a phosphorylation^19^.

We conducted a gene reporter assay to measure FOXO3a activity. The luciferase assay results, which align with those from the western blot analysis, indicate that Klotho amplifies FOXO3a activity under both low and high glucose conditions (Fig. 2B). FOXO3a, upon activation, produces several antioxidant proteins that play a crucial role in the cellular response to oxidative stress^53^. Importantly, we noted a dose-dependent increase in catalase expression subsequent to Klotho treatment (Fig. 2C).

Aging correlates with diminished FOXO3a expression, which subsequently results in abnormal astrocyte activation, inflammation, and metabolic disturbances^51^. Klotho, an antiaging protein, may potentially enhance FOXO-3a activity and catalase expression in astrocytes, thereby suggesting a possible protective mechanism.

In the subsequent phase of our research, we explored the potential of Klotho to protect neurons and astrocytes from cell death triggered by an oxidative insult, specifically H_2_O_2_. In astrocytes, Klotho pretreatment significantly mitigated H_2_O_2_-induced cell death (Fig. 3A,B). For glial cells, a concentration of 500 µM H_2_O_2_ constituted a moderate insult, while for neurons, the same concentration posed a severe insult (Fig. 3C). Consequently, pretreatment with an identical concentration of Klotho failed to safeguard neurons from H_2_O_2_-induced cell death (Fig. 3D).

Prior literature indicates that a 24-h pretreatment with Klotho can protect retinal pigment epithelial cells from H_2_O_2_-induced cell death. This protection is achieved by reducing cleaved-caspase 3 and Bax levels, and enhancing antioxidant activity, such as SOD2 and CAT^54^. These findings suggest that Klotho’s protective role against oxidative stress could be attributed to a decrease in AKT-mTOR signaling, which subsequently leads to an increase in FOXO3a activity and catalase levels. Our research further supports the hypothesis that Klotho functions as an anti-aging protein with antioxidant properties^54^.

The dependence on mitochondrial oxidative phosphorylation (OXPHOS) for survival significantly distinguishes astrocytic metabolism from neuronal metabolism. In astrocytes, OXPHOS inhibition triggers glycolysis, a response not mirrored to the same extent^55^. Neurons rely on OXPHOS to prevent apoptosis resulting from energy failure^56^. This reliance is partially attributed to the diminished levels of a key glycolytic-promoting enzyme, PFKFB3^57^.

PFKFB3, a crucial enzyme, plays a significant role in managing energy metabolism and antioxidant defenses in neurons. This enzyme belongs to the PFKFB family, which is tasked with controlling the concentration of fructose-2,6-bisphosphate (F2,6BP) within cells. F2,6BP serves as a powerful activator for the glycolytic enzyme PFK-1, the primary enzyme in the glycolytic pathway responsible for the conversion of glucose into pyruvate.

The levels of PFKFB3 messenger RNA in neurons and astrocytes are identical. However, owing to ubiquitination leading to proteasomal degradation, the protein level of PFKFB3 in neurons is virtually null due to its ubiquitination and consequent proteasomal degradation^41^. Maintaining low PFKFB3 levels in neurons can promote the PPP, a crucial process for the production of NADPH and ribose-5-phosphate. These compounds are essential for nucleotide and fatty acid synthesis, as well as for antioxidant defenses^58^.

Conversely, glutamate-induced excitotoxicity stabilizes neuronal PFKFB3. This stabilization subsequently diminishes the activity of the PPP and augments glycolysis, leading to oxidative damage and, ultimately, neuronal death. These detrimental effects can be mitigated by the overexpression of glucose-6-phosphate dehydrogenase, the enzyme that catalyzes the rate-limiting step in the PPP^59^. A reduction in PFKFB3 levels within neurons can enhance PPP activity and NADPH production^59,60^. In adipocytes, PFKFB3 activity is decreased by Klotho^39^.

In this study, we demonstrated that Klotho can diminish PFKFB3 levels in neurons (Fig. 4B) by promoting an upsurge in ubiquitination and proteasome activity (Fig. 5). This discovery is particularly significant as the accumulation of misfolded and damaged proteins is a characteristic feature of aging and is associated with neurodegenerative diseases^61^.

**Figure 6:**
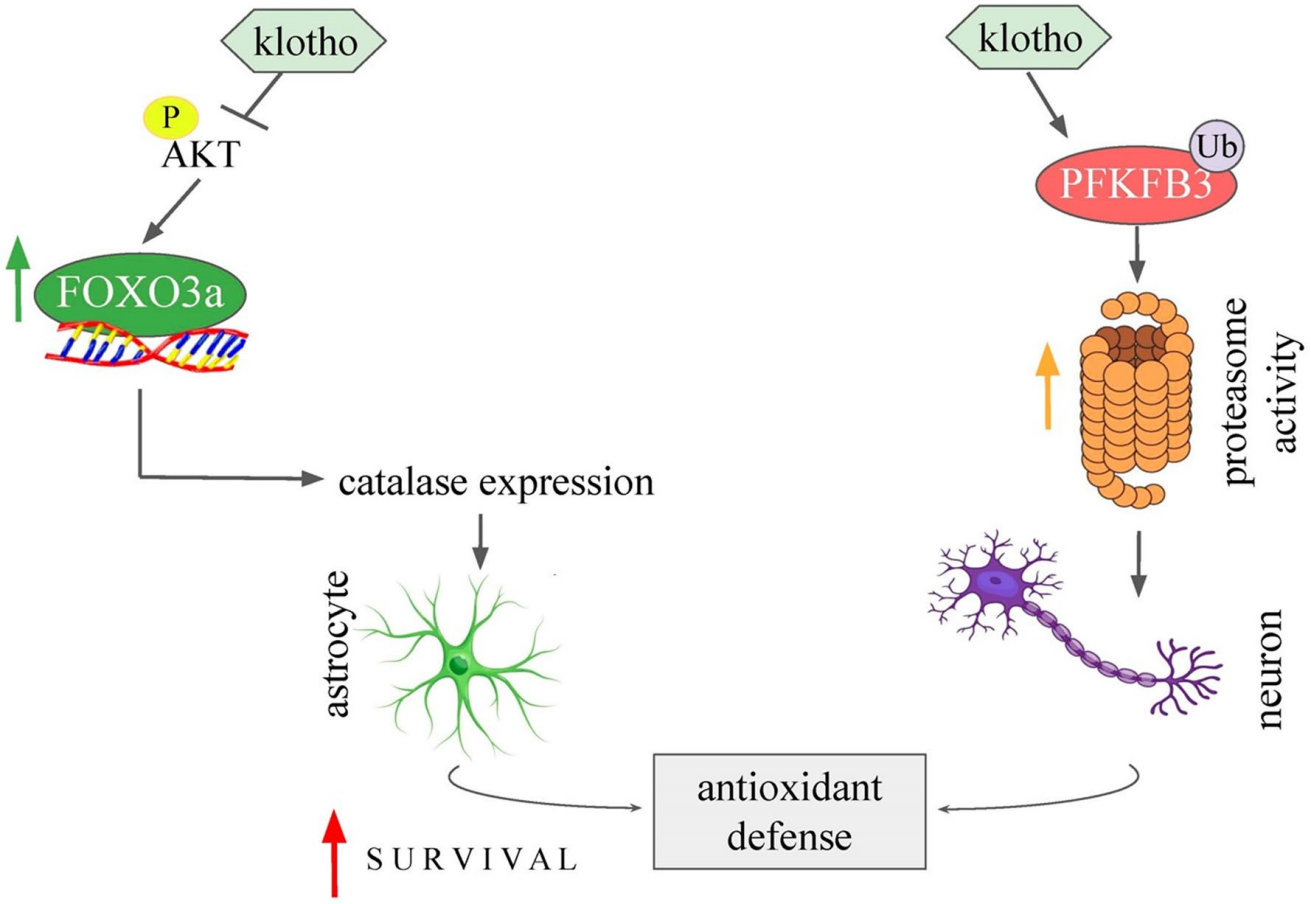
Schematic representation of the effects of Klotho in astrocytes and cortical neurons. The AKT inhibition by Klotho treatment induces transcriptional activity of FOXO transcription factors and promotes antioxidant defense in astrocytes by inducing catalase expression. In addition, Klotho treatment induced PFKFB3 ubiquitination and proteasome activity in neurons. Klotho is an important player in the adaptive defense response in astrocytes, and it increases proteasomal activity in neurons, which are both protective actions involving coupling between neurons and astrocytes against neurodegenerative processes.

In conclusion, our research offers novel insights into the functions of Klotho in controlling energy metabolism and the redox state within the brain. The data we have gathered indicates that Klotho inhibits insulin/IGF-1 signaling in neurons and functions as an antioxidant in astrocytes by increasing FOXO activity and promoting catalase expression. Additionally, Klotho reduces PFKFB3 content in neurons, a crucial factor in the regulation of NADPH levels and the overall cellular reducing potential. Our study highlights the interaction between energy metabolism and redox balance, proposing that Klotho could be instrumental in preserving this equilibrium (Fig. 6).

### Supplementary Information

Supplementary Figure S1.

Supplementary Figure S2.

Supplementary Figure S3.

Supplementary Figure S3.

Supplementary Figure S4.

Supplementary Figure S5.

Supplementary Figure S6.

Supplementary Figure S7.

## Data Availability

The datasets used and/or analyzed during the current study are available from the corresponding author on reasonable request.
