# Supplementary figures and images for: Klotho increases antioxidant defenses in astrocytes and ubiquitin–proteasome activity in neurons
Source: Sci Rep. 2023 Sep 12;13:15080. doi: 10.1038/s41598-023-41166-6 (PMC10497516; doi:10.1038/s41598-023-41166-6)

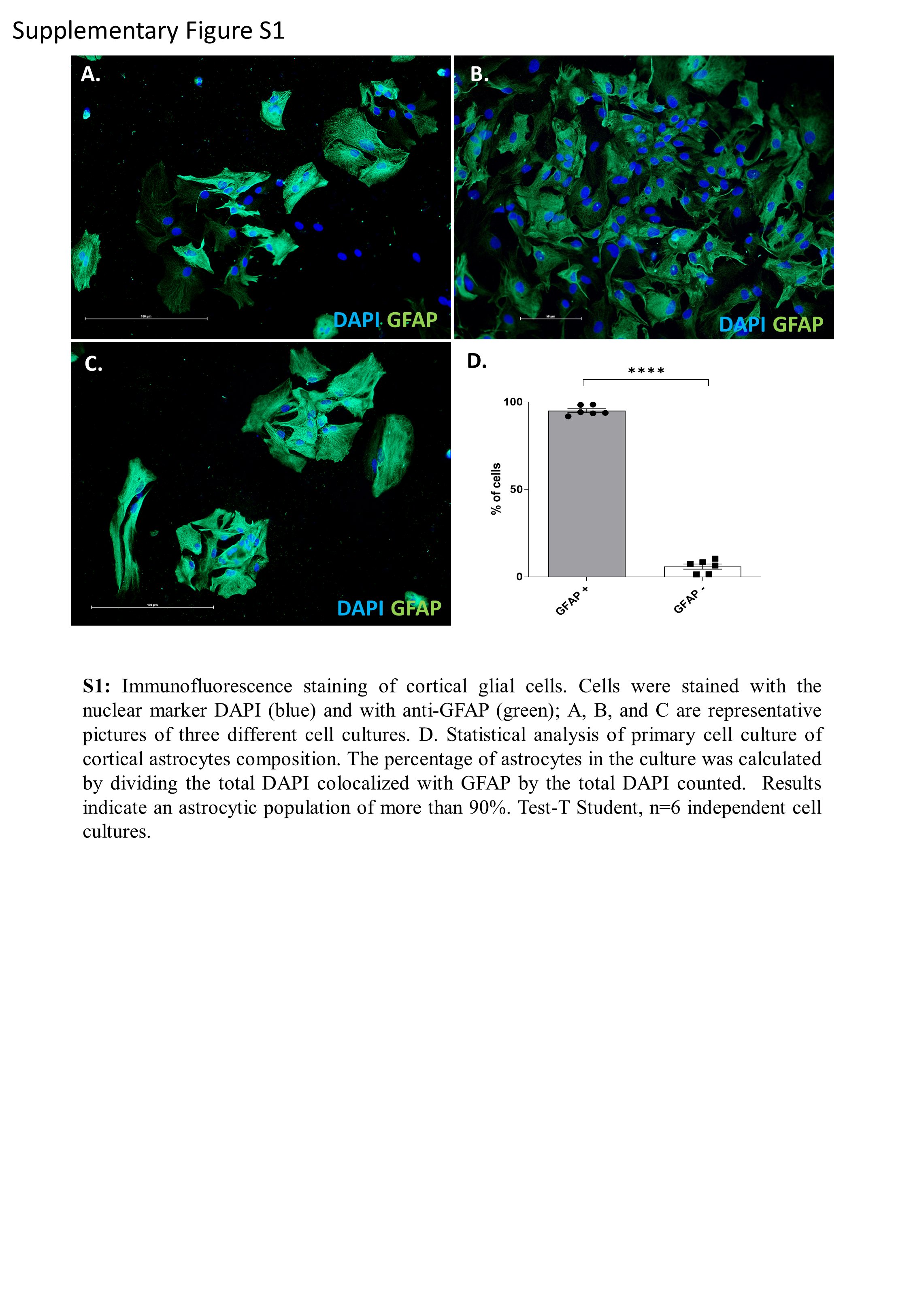

Supplement: Supplementary file 1 — Supplementary Figure S1. [file 41598_2023_41166_MOESM1_ESM.tif]

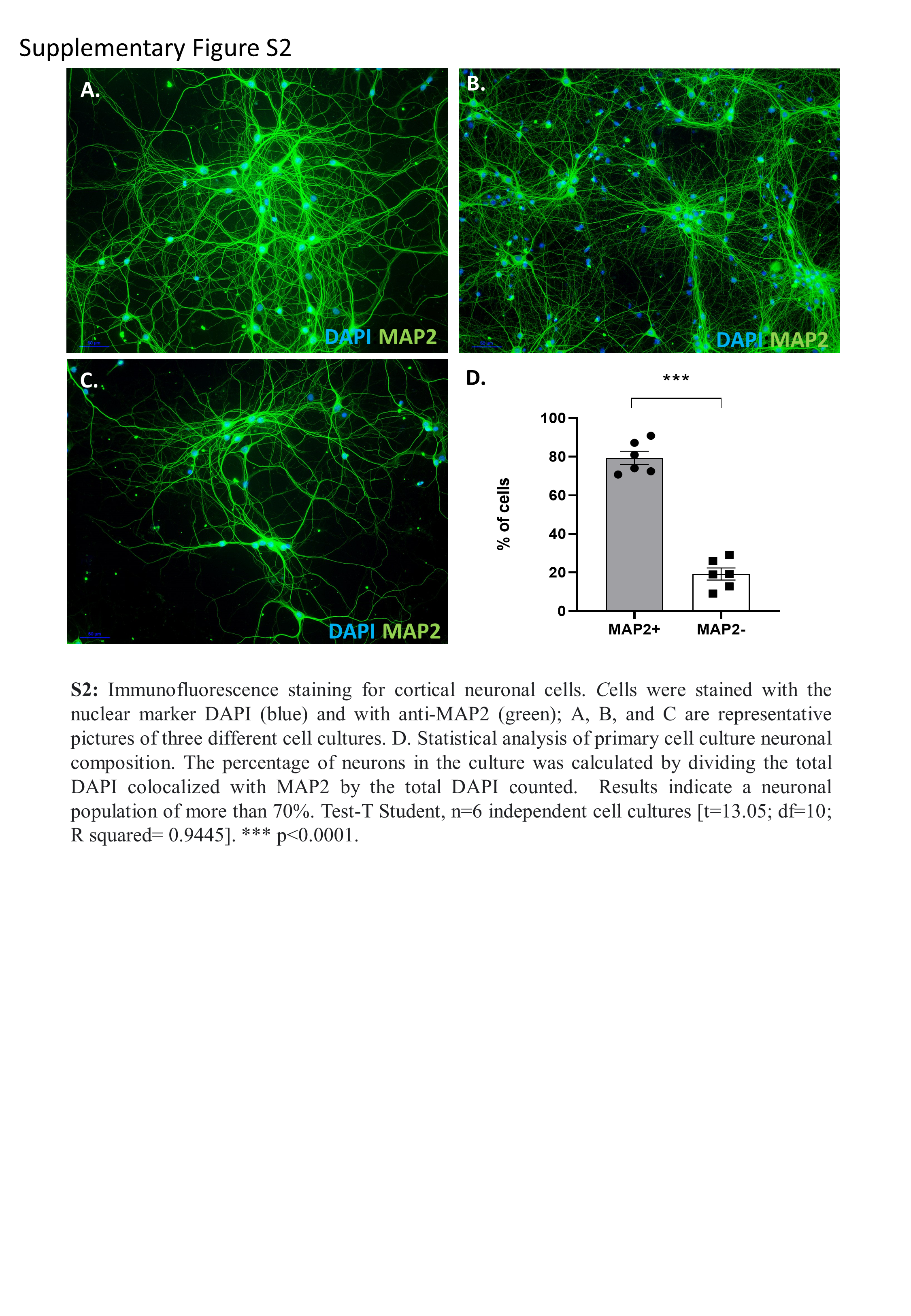

Supplement: Supplementary file 2 — Supplementary Figure S2. [file 41598_2023_41166_MOESM2_ESM.tif]

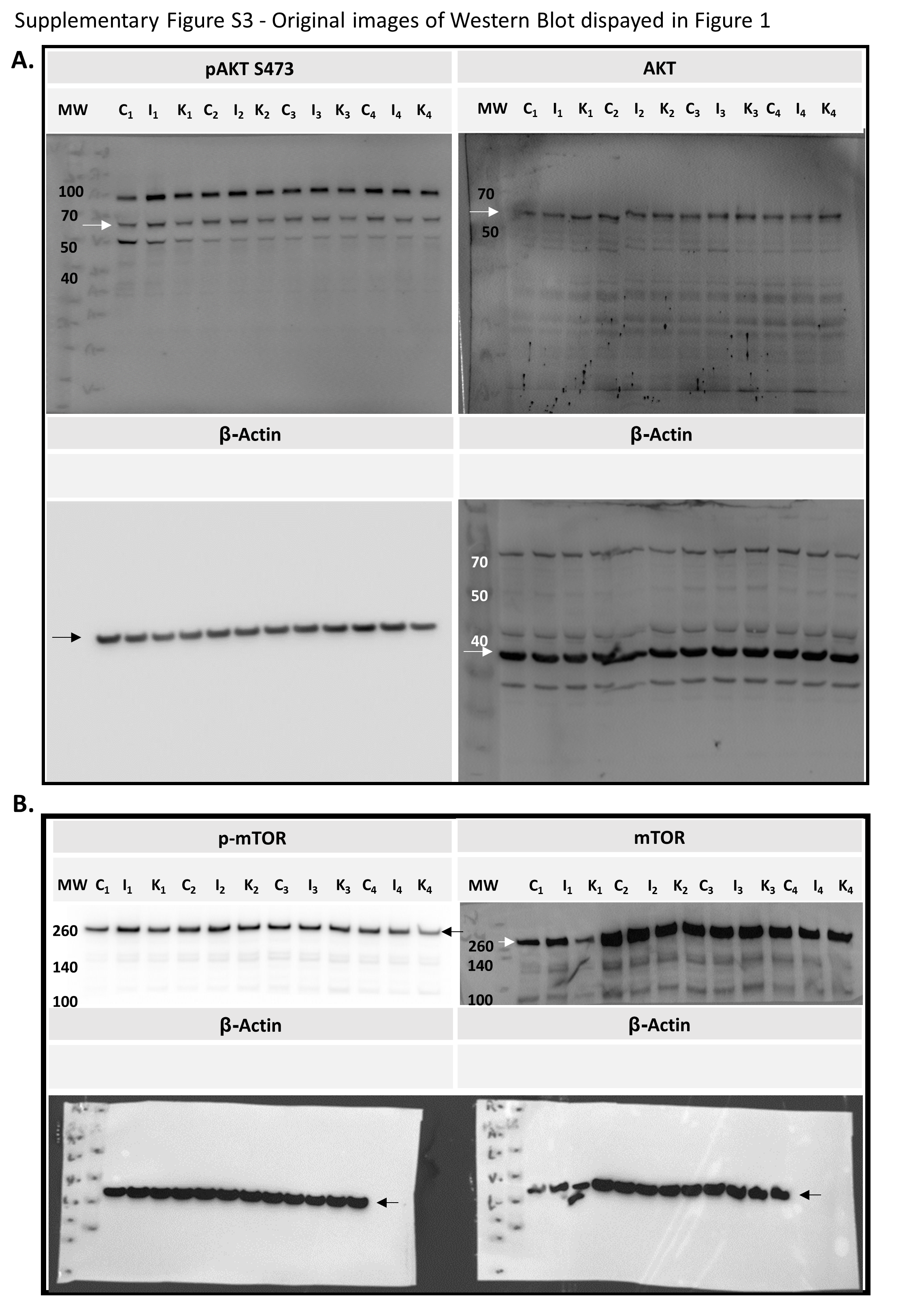

Supplement: Supplementary file 3 — Supplementary Figure S3. [file 41598_2023_41166_MOESM3_ESM.tif]

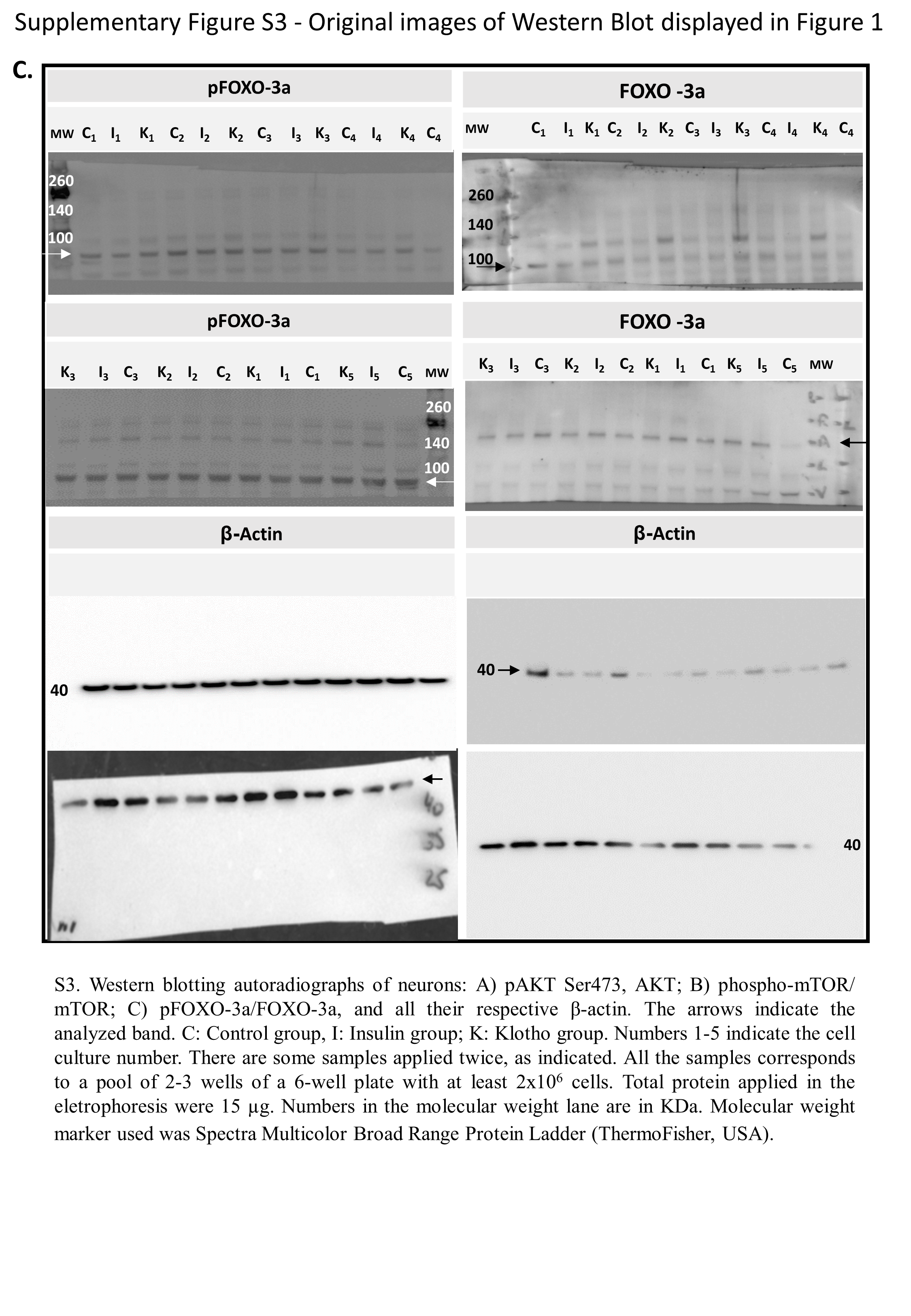

Supplement: Supplementary file 4 — Supplementary Figure S3. [file 41598_2023_41166_MOESM4_ESM.tif]

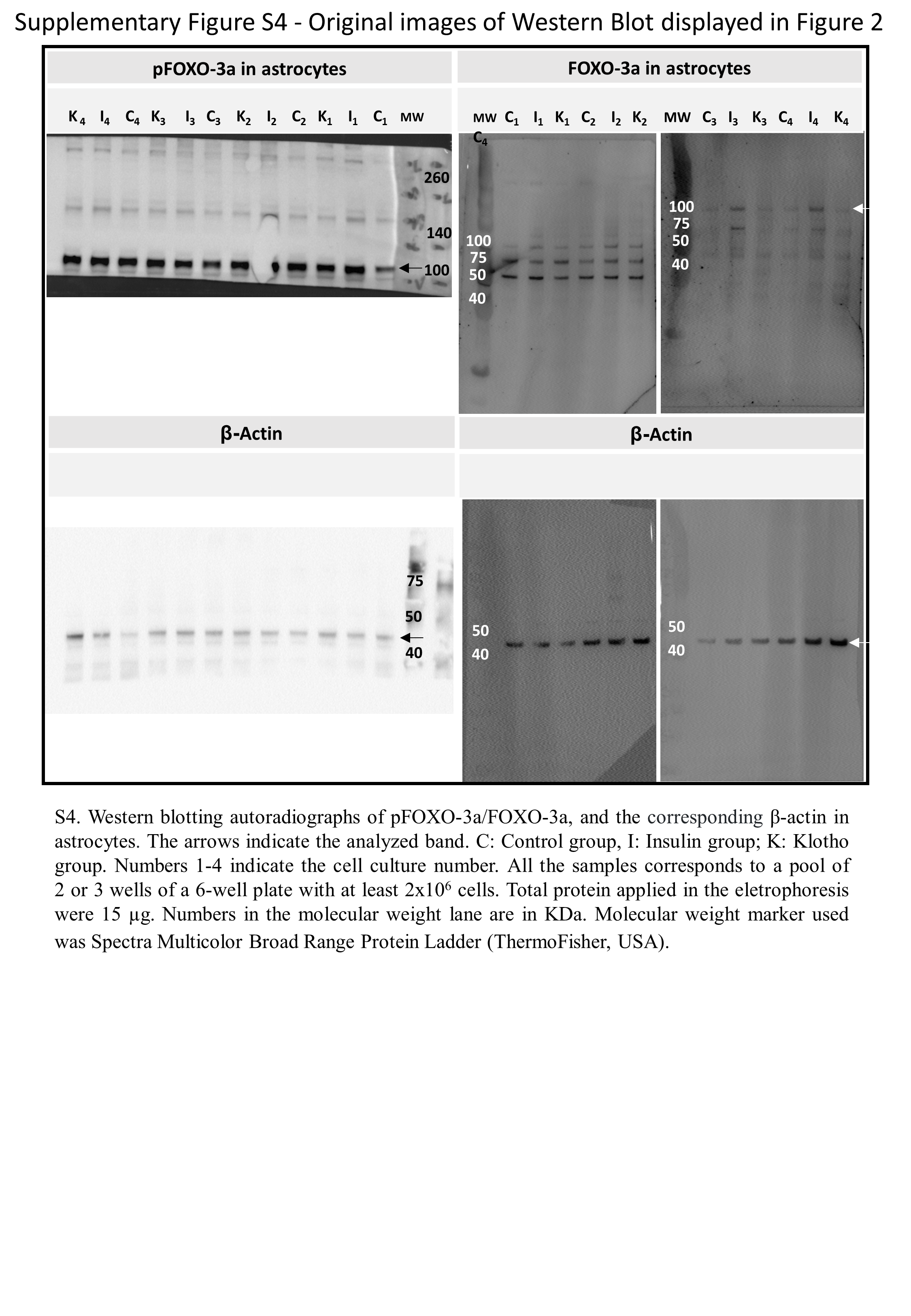

Supplement: Supplementary file 5 — Supplementary Figure S4. [file 41598_2023_41166_MOESM5_ESM.tif]

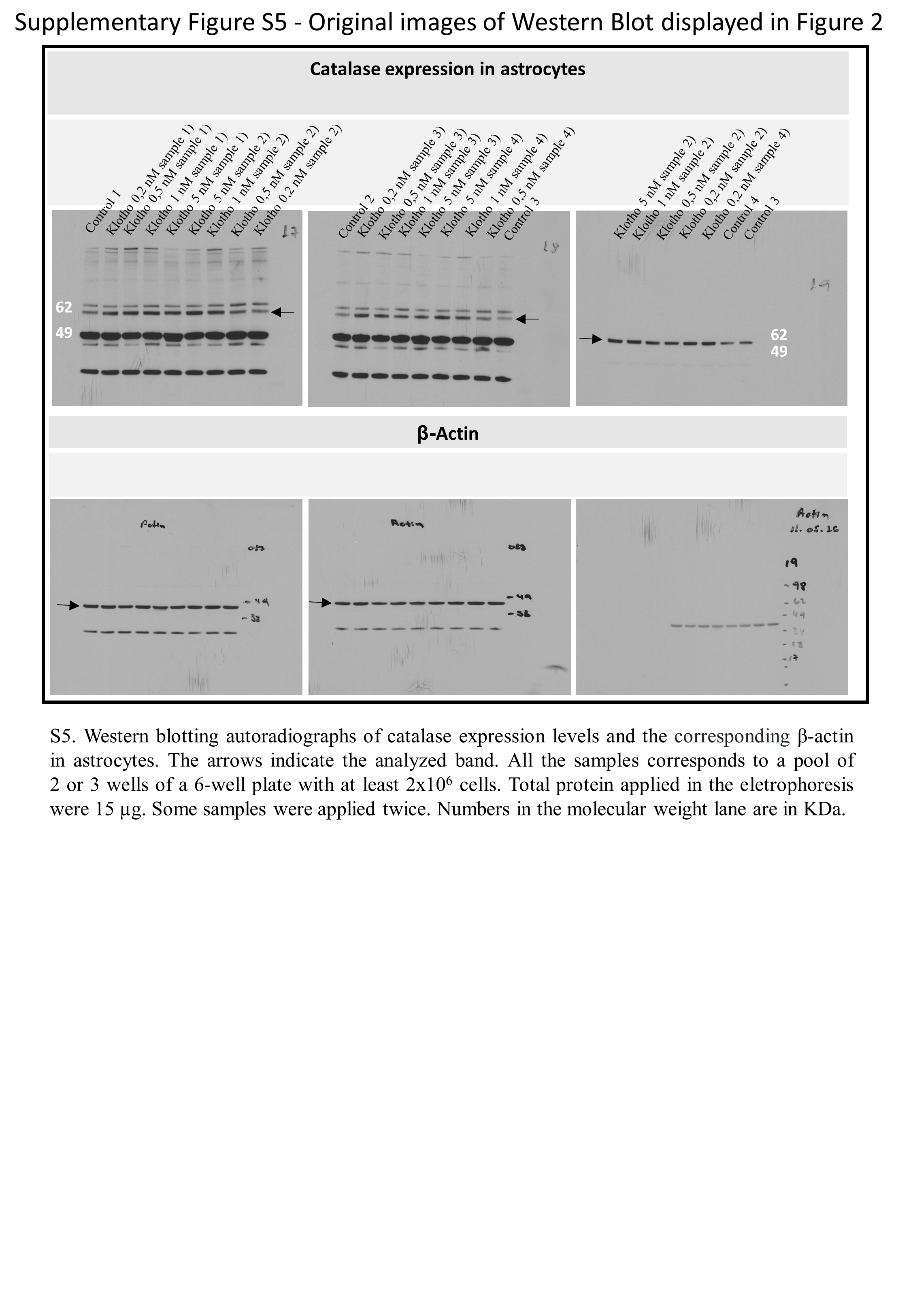

Supplement: Supplementary file 6 — Supplementary Figure S5. [file 41598_2023_41166_MOESM6_ESM.tif]

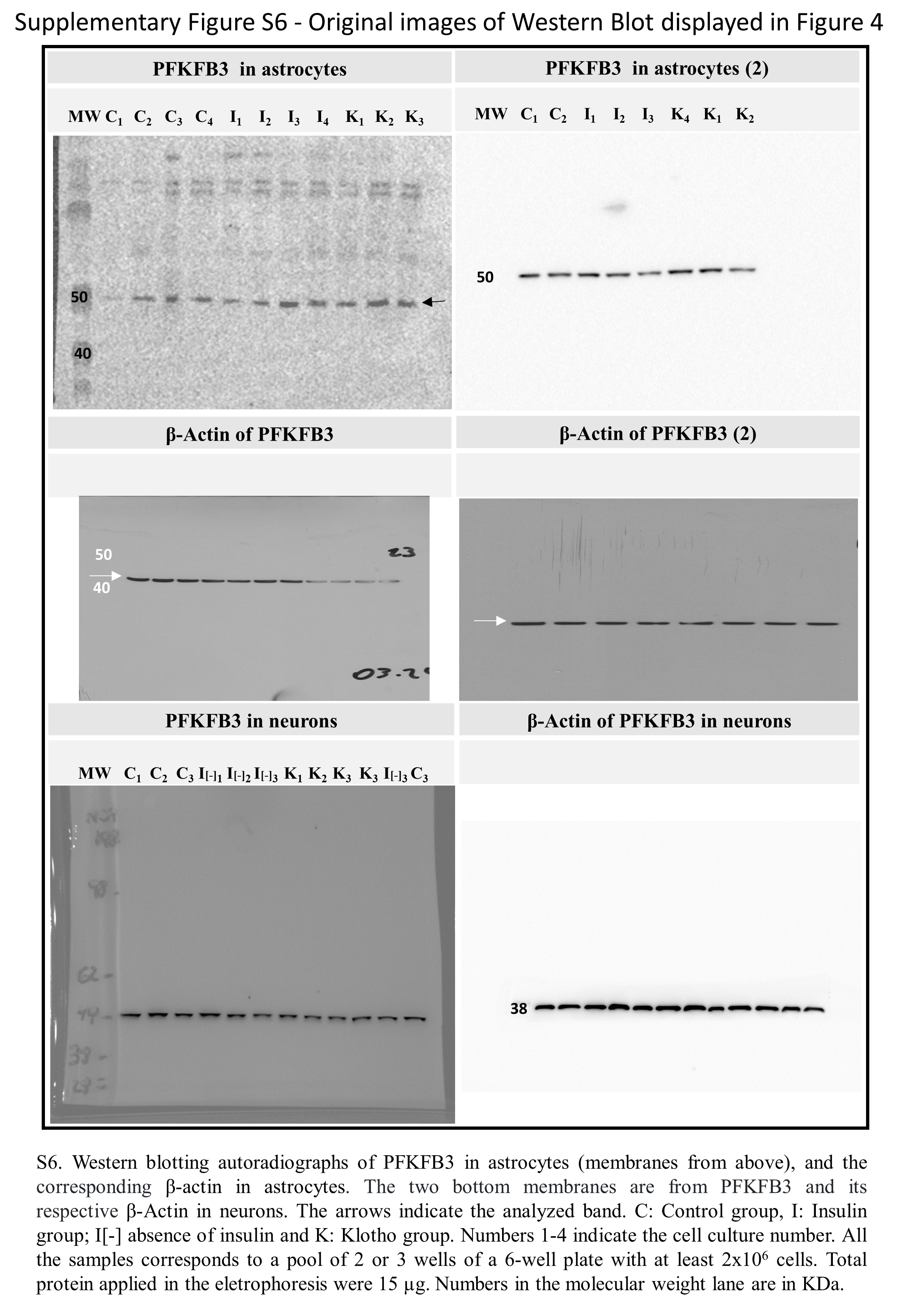

Supplement: Supplementary file 7 — Supplementary Figure S6. [file 41598_2023_41166_MOESM7_ESM.tif]

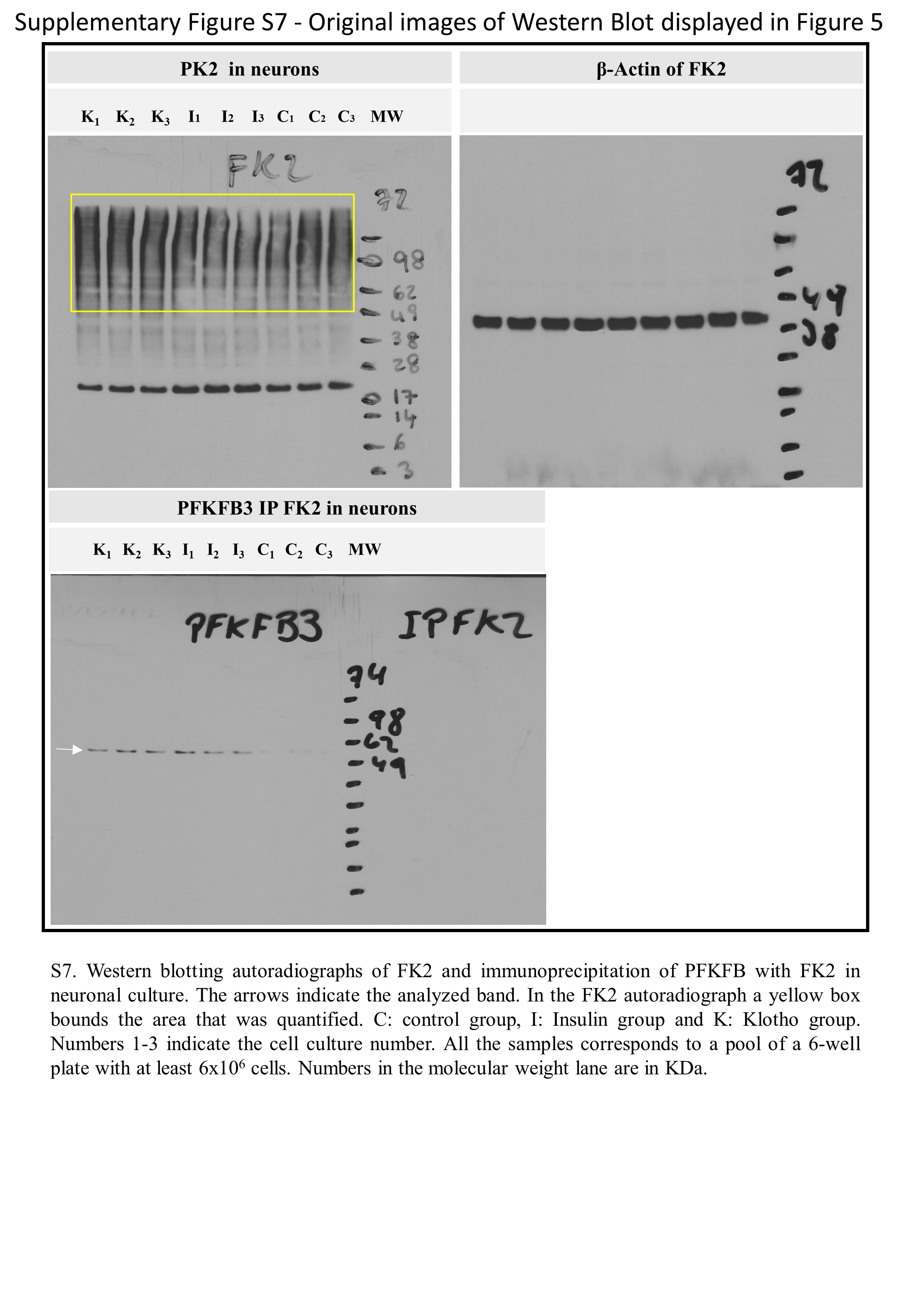

Supplement: Supplementary file 8 — Supplementary Figure S7. [file 41598_2023_41166_MOESM8_ESM.tif]
